# Evaluation of method performance for osteometric sorting of commingled human remains

**DOI:** 10.1080/20961790.2018.1535762

**Published:** 2019-02-07

**Authors:** John E. Byrd, Carrie B. LeGarde

**Affiliations:** Laboratory, Defense POW/MIA Accounting Agency, Joint Base Pearl Harbor-Hickam, HI, USA

**Keywords:** Forensic sciences, forensic anthropology, statistics, test method, method performance, error, signal detection, osteometric sorting

## Abstract

Evaluation of method performance involves the consideration of numerous factors that can contribute to error. A variety of measures of performance can be borrowed from the signal detection literature and others are drawn from statistical science. This article demonstrates the principles of performance evaluation by applying multiple measures to osteometric sorting models for paired elements run against data from known individuals. Results indicate that false positive rates are close, on average, to expected values. As assemblage size grows, the false positive rate becomes unimportant and the false negative rate becomes significant. Size disparity among the commingled individuals plays a significant role in method performance, showing that case-specific circumstances (e.g. assemblage size and size disparity) will determine method power.

## Introduction

Fundamental to forensic anthropology is the notion of method performance and error rates. While all scientific endeavours seek to produce replicable results based on rigorous and reliable procedures, forensic applications of science must be transparent and explicit about sources of error and how to mitigate it. This article is an examination of the nature of error in osteometric sorting of commingled remains. We will use statistical models for sorting paired elements as examples of how method performance can be conceptualized, measured, and to some degree controlled. The proximate purpose of the article is to provide fundamental measures of method performance for select osteometric models. The ultimate purpose is to demonstrate how method performance and error should be viewed in forensic anthropology.

We wish to be explicit about the terms we are using and the purposes of the various measures of error and performance adopted. A *method* is any procedure, technique or planned way of performing work. A *test method* or *test* is an analytical method defined by a specific protocol which is intended to produce a specific range of responses, including guidance on how the responses are to be interpreted. The test protocol includes details such as measurements to be taken, the level of precision required and the statistical models used to evaluate data. Test methods involving statistical analysis typically have recommended cutoff values (e.g. *P* < 0.10) or bifurcating guidelines (e.g. male if value >0).

Osteometric sorting, as we have promoted it, uses significance tests. Other approaches such as use of likelihood ratios can be valid as well. In practice, we have tended to use a cutoff value of *P* < 0.10, though have been adamant that practitioners should use whatever cutoff they find appropriate given the case they are working. Byrd and Adams [1] originally proposed 0.10 as a convenient cutoff for their work based on intuition and experience. An important point to bear in mind is that when using significance testing as a statistical approach, each *P* value obtained is to be interpreted in the context of the test method being used, including the circumstances of the case. (Note: significance tests are generally meant to support an inference informed by data, not serve as the singular finding of an experiment). How one views a *P* value of 0.07 in a case with two individuals commingled should not necessarily be the same as in a case with over 300 individuals commingled.

The above points notwithstanding, we need to cast osteometric sorting into a more draconian framework for the purposes of exploring error and performance rates. This means that for purposes of this study we will collapse the significance test results into a binary rule whereby any *P* value below the stated cutoff value is a “positive” result (rejecting the null hypothesis) and any *P* value above the cutoff value is a “negative” result (failing to reject the null hypothesis). For osteometric sorting, we will view the rejection of an association as positive (segregate them because they are disparate sizes and should not be from the same individual) and the failure to reject as negative (cannot segregate, but this does not preclude that they are from separate individuals). This exercise makes possible the employment of a battery of measures of performance, most of which are borrowed from the signal-detection world. One should bear in mind that collapsing significance tests into this format is a heuristic device meant to explore performance issues.

The following signal-detection related measures (as in [2]) will be used in this article to evaluate osteometric sorting tests for paired elements:

True positive rate (TPR) – Rate of obtaining a positive test result when the bones are known to be from separate individuals.

False positive rate (FPR) – Rate of obtaining a positive test result when the bones are known to be from the same individual.

True negative rate (TNR) – Rate of obtaining a negative test result when the bones are known to be from the same individual.

False negative rate (FNR) – Rate of obtaining a negative result when the bones are known to be from different individuals. (Note: osteometric sorting is not by design able to segregate bones from individuals of approximately the same size since the models focus generically on bone size).

Prevalence (P) – The proportion of comparisons of bones known to be from different individuals.

Level of test (Q) – The proportion of comparisons that produce a positive result (Q = TP + FP).

Sensitivity (SE) – The proportion of comparisons that are found to be TP out of all comparisons of bones known to come from different individuals (TP/P).

Specificity (SP) – The proportion of comparisons that are found to be TN out of all the comparisons of bones known to come from the same individual (TN/(1-P)).

Positive predictive value (PPV) – The proportion of comparisons that are found to be TP out of all comparisons that produced a positive test result (TP/Q).

Negative predictive value (NPV) – The proportion of comparisons that are found to be TN out of all comparisons that produced a negative test result (TN/(1-Q)).

Efficiency (EFF) – The overall correct classification rate, as in the number of TP and TN results divided by the overall number of comparisons. The overall error rate is 1 – Efficiency.

Sensitivity Quality Index (κ(1,0)) – A measure of the sensitivity in the context of the level of the test ((SE-Q)/(1-Q). This statistic takes into account the fact that if the level of the test is 99%, and the SE is 99%, that is not a very useful test since by calling every result positive, you guarantee high sensitivity and ignore a great many FPs.

Specificity Quality Index (κ(0,1)) – A measure of the specificity in the context of the level of the test ((SP-(1-Q))/Q). Like the κ(1,0), this statistic evaluates specificity against the level of test to guard against inflated specificity driven only by the design of the test (as in a level of test being 0.01 and nearly all comparisons are necessarily found to be negative, producing an inflated SP value).

In addition, we will explore the concept of the false discovery rate (FDR) in the context of osteometric sorting as described by Sorić [3], defined as below:

FDR (Q_actual_) – The observed proportion of the comparisons with a positive result that are not TP (in other words, 1-PPV). This proportion is derived in validation studies where the correct answer is known through independent means.

Maximum FDR (Q_max_) – The projected maximum number of positive results that are FP. This is an estimate relying upon model assumptions combined with observed results.

## Methods

The results from four different studies, each using different reference databases, are examined in light of the various measures of error and performance described above. The central study examined is that reported in Byrd and LeGarde [4], focusing on the models for paired elements. This study utilized the reference data described in the paper which was compiled at the Defense POW/MIA Accounting Agency (DPAA) Laboratory for the purpose of building general models for osteometric sorting. The second study was performed by LeGarde as part of her Master of Arts thesis and combined an independent sample from the Bass Collection at the University of Tennessee, Knoxville with the original DPAA Laboratory data [5]. The third study was performed by LeGarde and included an independent collection of data from Chiba University in Japan. These data were used as an independent check on the original Byrd and LeGarde models in this article. Finally, a fourth study was reported by Vickers et al. [6] and utilized data from the Forensic Databank of the University of Tennessee, Knoxville. (Note: we declined to consider Vickers et al. [6] treatment of archaeological data since it did not involve known individuals.) The results from all four studies permit the assessment of the FPRs when testing the Byrd and LeGarde models against the various reference data. FPRs from the four studies are provided in Tables 1–4. The results for Byrd and LeGarde studies were calculated from the respective databases in Microsoft Excel. The Vickers et al. [6] results were extracted from their original publication and pertain only to the Forensic Databank. Along with the FPR for each model is given the significance level for a binomial test comparing the FPR to the expected result of 0.10 (since the cutoff value used was *P* = 0.10, we test the null hypothesis that the FPR = 0.10). Finally, the mean FPR for the group of results is provided along with the probability that at least one of the *P* values should be significant at the 0.05 level (calculated as given in the table footnote).

**Table 1.** False positive results from the study of Vickers et al. [6] and application of performance metrics of Byrd models.

Source	Model	*N*	FPR	*P* (1-sided)	SP
[6]	Humerus	1 063	0.09	0.91	0.91
	Radius	981	0.12	0.05	0.88
	Ulna	934	0.17	1.6 × 10^−11^	0.83
	Femur	1 001	0.08	0.95	0.92
	Tibia	933	0.08	0.97	0.92
	Fibula	855	0.07	0.99	0.93
Mean FPR					0.10
*P* ≥ 1 significant result					0.47

FPR: false positive rate; SP: specificity

**Table 2.** False positive results from the study of Byrd and LeGarde [4] and application of performance metrics of Byrd models.

Source	Model	*n* + number of random pairings	FPR	*P* (1-sided)	Q_actual_	Q_max_	SE	SP	κ(0,0)	κ(1,0)	PPV	NPV	EFF
[4]	Humerus	188 + 997 = 1 185	0.05	0.99	0.01	0.04	0.84	0.95	0.93	0.44	0.99	0.52	0.86
	Radius	117 + 931 = 1 048	0.13	0.12	0.02	0.03	0.84	0.88	0.84	0.34	0.98	0.42	0.85
	Ulna	107 + 911 = 1 018	0.20	0.00083	0.03	0.03	0.84	0.80	0.75	0.30	0.97	0.37	0.84
	Humerus (no TL)	272 + 992 = 1 264	0.07	0.95	0.02	0.09	0.68	0.94	0.89	0.29	0.98	0.45	0.74
	Radius (no TL)	241 + 994 = 1 235	0.08	0.84	0.03	0.07	0.74	0.92	0.86	0.33	0.97	0.46	0.78
	Ulna (no TL)	63 + 886 = 949	0.08	0.61	0.01	0.06	0.67	0.92	0.88	0.11	0.99	0.17	0.69
Mean FPR													0.10
*P* ≥ 1 significant result													0.47

FPR: false positive rate; Q_actual_: false discovery rate; Q_max_: maximum false discovery rate; SE: sensitivity; SP: specificity; κ: sensitivity quality index; PPV: positive predictive value; NPV: negative predictive value; EFF: efficiency; TL: total length of bone: UT: University of Tennessee

**Table 3.** False positive results from the study of LeGarde [5] and application of performance metrics of Byrd models.

Source	Model		*N*	FPR^a^	*P* (1-sided)	SP
[5] (UT data)	Humerus	HML	151	0.07	0.84	0.93
		HEB	148	0.13	0.10	0.87
		MDDT	151	0.13	0.12	0.87
		HML + MDDT	151	0.09	0.66	0.91
		HML + MDDT + HEB	148	0.09	0.63	0.91
		HML + MDDT + HEB + HMiD	148	0.10	0.52	0.91
		MDDT + HMiD	151	0.13	0.12	0.87
	Radius	RML	142	0.10	0.45	0.90
		MDRT	145	0.08	0.70	0.92
		RML + MDRT	142	0.13	0.12	0.87
		MDRT + RMiD + RMaD	144	0.12	0.19	0.88
	Femur	FML	119	0.07	0.85	0.93
Mean FPR						0.10
*P* ≥ 1 significant result						0.71

FPR: false positive rate; SP: specificity; UT: University of Tennessee

a Using standard deviation calculated from the sample (for the UT data test, the sample and reference data together produced the standard deviation), rather than Byrd standard deviation.

**Table 4.** False positive results from the study of LeGarde [5] and application of performance metrics of Byrd models.

Source	Model	*N*	FPR		*P* (1-sided)		SP
[5] (Chiba)	Humerus	46	0.13,	0.04	0.17,	0.85	0.87 (0.96)
	Radius	47	0.15,	0.09	0.09,	0.51	0.85 (0.91)
	Ulna	23	0.13,	0.04	0.19,	0.68	0.87 (0.96)
	Femur	46	0.07,	0.02	0.69,	0.95	0.93 (0.98)
	Tibia	46	0.04,	0.02	0.85,	0.95	0.96 (0.98)
	Fibula	43	0.05,	0.05	0.82,	0.82	0.95 (0.95)
Mean FPR						0.09	0.04
*P* ≥ 1 significant result							0.47

FPR: false positive rate; SP: specificity.

None of these studies permit examination of the more interesting question as to the overall performance of the models. This assessment requires one to measure the test accuracy when bones known not to be associated (i.e. from the same individual) are compared along with the accuracy for those that are associated. To facilitate this fuller assessment, bone data from the DPAA Laboratory database were randomly commingled and compared to one another. The procedure for random pairing was 1) assign a number to each individual, 2) pair right and left bones for comparison by assigning random numbers for the left and right bones to be compared 1 000 times, 3) delete all instances of two bones from the same individual since results concerning performance against known matches already existed. For easy reference in this article, this exercise will be referred to as the “Byrd study”. The *t*-tests using the Byrd and LeGarde models were performed on the random pairings. All of these steps were performed in Microsoft Excel. The simulated comparisons of bones from different individuals were combined with the comparisons from the same individuals to produce a more complete suite of performance metrics in Tables 1–4. The metrics in Tables 1–4 were calculated in Microsoft Excel according to the definitions provided above.

While it is clear that performance metrics for correctly segregating bones depend greatly upon the size disparity of the individuals to be compared, it is nonetheless interesting to project performance in future applications under the assumption that size disparity will be effectively the same as seen in the DPAA Laboratory data. This is not a “safe assumption”. One issue to consider is that DPAA data include a sizable representation of healthy, young adult males. However, this exercise provides the reader a baseline against which to base expectations in casework. To the extent that the data used here reflect a variety of body sizes and the simulation described above utilized random pairings, the FPRs and FNR’s can be considered “average”. Table 5 provides expected PPVs and NPVs for commingled assemblages as small as 2 and as large as 400 individuals for the Byrd and LeGarde models that include the total lengths. All calculations rely on the FPR and NPR from the Byrd and LeGarde data resulting from the simulation described above. Calculations were performed in Microsoft Excel.

**Table 5.** Projections for future performance given performance metrics reported above for humerus, radius, and ulna (including TL).

Items	Sample			PPV			NPV		
	*N* (indiv)^a^	*N* (comp)^b^	Prev^c^	Humerus	Radius	Ulna	Humerus	Radius	Ulna
Projections	2	4	2	0.94	0.87	0.81	0.86	0.85	0.83
	3	9	6	0.97	0.93	0.89	0.75	0.74	0.71
	4	16	12	0.98	0.95	0.93	0.66	0.66	0.63
	5	25	20	0.99	0.96	0.94	0.60	0.59	0.56
	6	36	30	0.99	0.97	0.95	0.54	0.54	0.50
	7	49	42	0.99	0.98	0.96	0.50	0.49	0.45
	8	64	56	0.99	0.98	0.97	0.46	0.45	0.42
	9	81	72	0.99	0.98	0.97	0.43	0.42	0.38
	10	100	90	0.99	0.98	0.97	0.40	0.39	0.36
	11	121	110	0.99	0.98	0.98	0.37	0.37	0.33
	12	144	132	0.99	0.99	0.98	0.35	0.35	0.31
	13	169	156	1.00	0.99	0.98	0.33	0.33	0.29
	14	196	182	1.00	0.99	0.98	0.31	0.31	0.28
	15	225	210	1.00	0.99	0.98	0.30	0.29	0.26
	16	256	240	1.00	0.99	0.98	0.28	0.28	0.25
	17	289	272	1.00	0.99	0.99	0.27	0.27	0.24
	18	324	306	1.00	0.99	0.99	0.26	0.25	0.23
	19	361	342	1.00	0.99	0.99	0.25	0.24	0.22
	20	400	380	1.00	0.99	0.99	0.24	0.23	0.21
	30	900	870	1.00	0.99	0.99	0.17	0.17	0.15
	50	2 500	2 450	1.00	1.00	1.00	0.11	0.11	0.09
	70	4 900	4 830	1.00	1.00	1.00	0.08	0.08	0.07
	90	8 100	8 010	1.00	1.00	1.00	0.06	0.06	0.05
	100	10 000	9 900	1.00	1.00	1.00	0.06	0.06	0.05
	110	12 100	11 990	1.00	1.00	1.00	0.05	0.05	0.04
	120	14 400	14 280	1.00	1.00	1.00	0.05	0.05	0.04
	160	25 600	25 440	1.00	1.00	1.00	0.04	0.04	0.03
	200	40 000	39 800	1.00	1.00	1.00	0.03	0.03	0.02
	300	90 000	89 700	1.00	1.00	1.00	0.02	0.02	0.02
	400	160 000	159 600	1.00	1.00	1.00	0.01	0.01	0.01

a The number of individuals, each with a pair of elements.

b The number of comparisons calculated as *N* (indiv)^r^.

c Prevalence defined as number of pairwise comparisons NOT from same individual.

TL: total length of bone; PPV: positive predictive value; NPV: negative predictive value

## Results

Tables 1–4 show the FPRs for the various studies. While the FPRs for individual tests vary between 0.02 and 0.20, the mean FPRs are all 0.10 – the expected value – except for the LeGarde Chiba study. LeGarde conducted this study using the Byrd and LeGarde models and summary statistics and then also ran the tests using the mean and standard deviations from that skeletal group (all Japanese). (Recall that Byrd and LeGarde provided a standard deviation for each model that tests the null hypothesis as deviation from “0”). When she substituted the new summary statistics into the models she saw a dramatic drop in error rates, ranging from 0.02 to 0.09. On the whole, the results follow what one expects when statistical models are developed from one sample and then applied to others: variation in detailed outcomes but show a mean error close to the expected value. Even the results reported by Vickers et al. [6] show a mean error of 0.10. It is comforting to see the mean FPR close to or equal to 0.10 in the studies, but troubling to see that radius and ulna models that include the total length measurements perform consistently worse than expected (more on this issue later in the article).

Tables 1–4 go beyond the FPR and looks at the larger suite of performance metrics. Specificity can be provided for all of the studies and the values range from 0.80 to 0.96. The values vary for the different models in each study and there appear to be no striking differences between studies. Owing to the simulation through random pairings, the Byrd study shows all of the performance metrics. These are discussed further below.

Table 5 provides what might be considered projections of future performance under conditions where the size disparities of the commingled individuals are similar to the DPAA reference data. These projections are estimated using the FPRs and NPRs captured in the results shown in Tables 1–4. Only the models for complete bones (models include total lengths) were used in this exercise. This exercise is intended to be illustrative of key concepts, not a reliable predictor of future error rates in any particular case (see below). The obvious pattern to observe in the results is the diminishing values of NPV as the assemblage size grows beyond five commingled individuals. At more than six commingled individuals it is more likely than not that the bones are from different persons even when the test result is to accept the null hypothesis. We expect this pattern to always characterize osteometric sorting, even if the specific values of the NPV will vary from case to case due to varying size disparities of the commingled individuals. This is why all of our papers state emphatically that more confidence is to be placed in exclusions (results that indicate rejection) than the reverse. In larger assemblages, the failure to reject an association does not imply the bones must originate in the same person. Rather, it only indicates the bones originated from one person OR two persons of a similar size. Osteometric sorting is a method of exclusion when considered in isolation from other evidence.

## Discussion

The performance of osteometric pair-matching is influenced deeply by two factors: the size of the commingled assemblage and the size disparity among the commingled individuals. These two factors will vary from case to case. Thus, the power of the method will always be case-specific. Other factors, such as choice of statistical model (e.g. significance testing versus likelihood ratio approach) pale next to these two factors in determining overall performance. Table 6 illustrates the effect of size disparity by showing the FNR from comparisons of bones from separate individuals who were 4, 6 and 8 inches different in stature, respectively (using *P* = 0.10 as cutoff). This shows that individuals four or more inches apart correctly sorted apart >94% of the time. When these levels of size disparity are found in small (<6 individuals) assemblages, we should expect to achieve a high degree of accurate sorting using size alone. When assemblages are large (*N*> 6) and the size distribution of the commingled individuals is similar to that seen in the reference data used to calculate the statistical models, then we expect the error rates to fall on average very close to the expected error rates projected by the statistical model (e.g. observed error rate of 10% when using a cutoff value of *P* = 0.10). To the extent that the reference data we have used to calculate models is “average” in terms of size distribution, then the error rates projected by the statistical models are expected to be observed in many cases.

**Table 6.** Showing the effects of difference in body size (as represented by stature) on test performance.

Model	*N*	FNR		
		Δ = 4 inches	Δ = 6 inches	Δ = 8 inches
Humerus	437	0.06	0.03	0.03
Radius	407	0.05	0.02	0.01
Ulna	367	0.05	0.01	0.01

FNR: false negative rate

**Figure 1.**
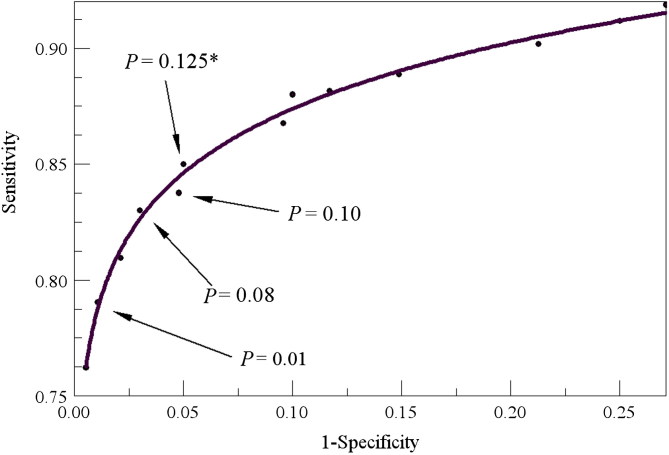
Receiver operating characteristic (ROC) curve for the humerus paired element model (with total length) with various *P* value cutoffs identified. The optimal value is *P*= 0.125.

It is clear that understanding the performance of a test method is far more complicated than the simplistic view espoused in Vickers et al. [6]. The most grievous shortcoming of that study is the limitation of error consideration to only the FP rates. Application of osteometric sorting in casework necessitates concern for the fuller suite of performance metrics presented in the tables above. What we see, for example, in Table 5 is that the PPV quickly rises to 1.0 as the size of the commingled assemblage gets larger. We also understand that case size disparity greater than that seen in the reference data used to calculate the statistical models will lead to better than projected performance. Thus, there is no good reason to optimize the test method to minimize the FP rate while ignoring other factors. Indeed, application of the cutoff values recommended by Vickers et al. [6] is expected to yield an overall poor performance as shown in Table 7. One way to view the optimal cutoff value is through receiver operating characteristic (ROC) curves [2]. Figure 1 shows a ROC curve for the humerus model (including total length). Given that the “ideal” or “optimal” cutoff value is the one whose performance levels plot closest to the upper left corner, it appears that *P* = 0.125 would be the best choice for cutoff. The solution recommended by Vickers et al. [6] is off the chart on the lower left corner and is clearly not optimal by this standard. While it is true that the risk of an FP is fully mitigated by the Vickers et al. [6] approach, it is done at unreasonable cost to overall method performance. We do not recommend this approach. As a final note on their study, we observed high error rates with the radius and ulna that are consistent with what Vickers et al. [6] reported for the Forensic Databank. It appears that there is systematic error for these elements that relates to varying degrees of curvature of the bones of the forearm. This topic is worthy of further study.

**Table 7.** Performance metrics for Vickers et al. [6] recommended approach applied to Byrd data.

Model	*N*	Standard [D](mm)	FNR	SE	SP	PPV	NPV	EFF
Humerus	188 + 997	0–31	0.55	0.45	1	1	0.45	0.54
Radius	117 + 931	0–23	0.56	0.44	1	1	0.44	0.50
Ulna	107 + 911	0–25	0.61	0.39	1	1	0.39	0.46
Humerus (no TL)	272 + 992	0–2.3	0.56	0.44	1	1	0.44	0.56
Radius (no TL)	241 + 994	0–3.6	0.41	0.44	1	1	0.59	0.67
Ulna (no TL)	63 + 885	0–5.1	0.57	0.43	1	1	0.43	0.47

FNR: false negative rate; SE: sensitivity; SP: specificity; PPV: positive predictive value; NPV: negative predictive value; EFF: efficiency; TL: total length

Another interesting study by Lynch et al. [7] demonstrates the effects of assemblage size on the FPR. Lynch developed computer simulations of comparisons by, for example, conducting *t*-tests of all possible pairwise associations of paired elements in the Forensic Databank sample. Using the Byrd and LeGarde [4] models, he made approximately 100 000 tests for each element. The FPRs, calculated as a proportion of all comparisons (not just comparisons where the bones were from the same individual), was less than 0.1% in every instance.

## Conclusion

The evaluation of method performance does not involve a single measure. Rather, one must consider a variety of factors that can be measured and/or assessed using results from validation studies. Application of the methods to known subjects where the correctness of the conclusions is independently determined provides a basis for understanding the various types of errors that can be encountered and how they might be mitigated. Osteometric sorting lends itself to such evaluation because the models can be turned back onto reference data comprised of known individuals and applied to independent samples. What is apparent in the results examined here is that the greatest determinant of the method’s performance will be the nature of the case it is applied to. The method(s) is at its best when the assemblage size is small and the size disparity of the commingled individuals is high. We expect that the performance over many cases to approximate what the statistical models project, combined with the assemblage size factors discussed above. We leave it to each person applying the methods to choose their optimal cutoff given the circumstances of the case.

## Compliance with ethical standards

This article does not contain any studies with human participants or animals performed by any of the authors.
